# Quantification of pre-existing radiographic damage and its relationship with joint activity and long-term clinical outcomes with secukinumab therapy in patients with psoriatic arthritis

**DOI:** 10.1186/s13075-022-02944-1

**Published:** 2022-12-28

**Authors:** Philip Mease, Désirée van der Heijde, Bruce Kirkham, Georg Schett, Ana-Maria Orbai, Christopher Ritchlin, Joseph F. Merola, Luminita Pricop, David A. James, Xuan Zhu, Gregory Ligozio

**Affiliations:** 1 Swedish Medical Centre, Providence St Joseph Health and University of Washington, 601 Broadway, Suite 600, Seattle, WA 98122 USA; 2 Leiden University Medical Centre, Leiden, the Netherlands; 3 Guy’s & St Thomas’ NHS Foundation Trust, London, UK; 4 Friedrich Alexander University Erlangen-Nuremberg and Universitätsklinikum Erlangen, Erlangen, Germany; 5 John Hopkins Arthritis Center, Baltimore, MD USA; 6 University of Rochester, Rochester, NY USA; 7 Brigham and Women’s Hospital, Harvard Medical School, Boston, MA USA; 8 Novartis Pharmaceuticals Corporation, East Hanover, NJ USA

**Keywords:** Psoriatic arthritis, Radiographic damage, Bone erosion, Joint space narrowing, Minimal disease activity

## Abstract

**Background:**

Psoriatic arthritis (PsA) patient data from two phase 3 secukinumab trials (FUTURE 1, 5) were analysed to quantify the prevalence and extent of pre-existing radiographic damage (RD) at baseline; investigate the association of RD with swollen/tender joint counts (SJC/TJC) at baseline; and investigate the extent to which RD at baseline correlated with response to secukinumab.

**Methods:**

Pooled data (*N* = 1554) provided baseline radiographic bone erosion and joint space narrowing (JSN) scores at pre-specified locations per the van der Heijde-modified total Sharp score (vdH-mTSS) for PsA and swollen and tender joint scores in the same joints at multiple visits. Overall patient RD and individual joints RD bone erosion and JSN scores were assessed. The association between joint activity (tenderness, swelling) and vdH-mTSS was assessed at the overall patient-level and individual joint tender, swollen scores (yes/no) and RD joint JSN and bone erosion scores at the individual joint-level. Treatment response was assessed using SJC/TJC at weeks 16 and 52 and the proportion of patients achieving minimal disease activity (MDA) over all assessments within 1 year from FUTURE 5 alone.

**Results:**

A substantial prevalence of pre-existing RD with higher prevalence of erosion than JSN was observed (86% and 60% of patients had positive erosion and JSN scores, respectively); higher RD prevalence was associated with longer time since PsA diagnosis. Joint activity was weakly associated with RD at baseline at the patient-level (Pearson’s coefficients: range 0.12–0.18), but strongly associated at the individual joint-level, with a higher probability of tender/swollen joints to associate with higher JSN/erosion scores: all 42 analysed joints showed statistical significance at the 0.05 level (unadjusted) for the relationship between joint tenderness (yes/no) and its JSN score, all but one for tenderness and bone erosion scores, and all but 2 for swollen and JSN scores and for swollen and bone erosion score. Secukinumab (150/300 mg), reduced TJC and SJC across all values of baseline erosion and JSN scores at weeks 16 and 52. Patients with higher levels of RD were less likely to achieve zero tender/zero swollen joint status and had lower chance of achieving MDA.

**Conclusions:**

PsA patients showed substantial prevalence of RD at baseline that correlated with time since diagnosis, but patient’s individual joint activity was strongly associated with pre-existing RD at those joints. Patients with the highest RD at baseline had a reduced likelihood of achieving zero joint count status.

**Supplementary Information:**

The online version contains supplementary material available at 10.1186/s13075-022-02944-1.

## Background

Psoriatic arthritis (PsA) is an inflammatory disease characterised by arthritis, enthesitis, and dactylitis, including spondylitis, skin, and nail psoriasis [1–3]. The characteristic radiographic features of PsA are bone erosions, joint space narrowing (JSN), bony proliferation, osteolysis, ankylosis, and formation of osteophytes [4].

Assessment of the extent of radiographic joint damage and progression is important in clinical trials evaluating treatments for PsA. Although overall joint damage is known to relate to physical function and quality of life (QoL), the specific contribution of individual bone erosion or narrowing changes to symptoms such as pain and response to therapy at the joint level are unclear [5, 6].

Secukinumab, a fully human monoclonal antibody that selectively inhibits interleukin (IL)-17A, demonstrated significant and sustained improvements in signs and symptoms in patients with PsA in phase 3 trials [7–11] and reduced radiographic progression in these patients [11, 12]. The results from the FUTURE 1 (NCT01392326) study revealed that subcutaneous (s.c.) secukinumab 150 mg with an intravenous loading dose effectively inhibited radiographic progression and demonstrated inhibition of scores for erosion and JSN in patients with active PsA up to 52 weeks. Radiographic benefits following initiation of secukinumab therapy were noted as early as week 24, irrespective of previous tumour necrosis factor inhibitor (TNFi) treatment [12].

The FUTURE 5 (NCT02404350) trial reported that secukinumab 300 and 150 mg, with or without a loading dose, significantly inhibited radiographic progression, in patients with PsA at week 24 with low rates of progression over 2 years [11].

In-depth patient-level and joint-level analyses of pooled data from these two phase 3 secukinumab trials were performed (1) to quantify the prevalence and magnitude of pre-existing radiographic damage (RD) at baseline; (2) to investigate the association between RD and clinical swollen (SJC) and tender joint count (TJC) prior to secukinumab therapy; and (3) to investigate the extent to which RD at baseline could influence the response to secukinumab therapy at weeks 16 and 52.

## Methods

### Patients and assessments

Pooled data from 1554 patients with PsA from the FUTURE 1 and FUTURE 5 studies were included for all baseline analyses and to assess the association of RD with joint activity prior to initiating secukinumab therapy. Data from FUTURE 5 (*N* = 987) with approved secukinumab s.c. doses (150 mg without load [NL], 150 mg with load, and 300 mg) were used to assess the extent to which RD at baseline could influence the response to therapy. Details of the study design and inclusion and exclusion criteria of both studies have been reported previously [7, 11].

Scoring of radiographic images was centrally performed by two independent readers who were blinded to all patient information, allocation of therapy and chronology of radiographs. If adjudication was needed, a third reader, i.e., an adjudicator who is different from the two primary independent reviewers, conducted an independent assessment. An adjudicator was required if there was a discrepancy of ≥ 8 units in van der Heijde-modified total Sharp score for PsA (vdH-mTSS) change from baseline. The average score of two readers was used in all analyses.

**Fig. 1:**
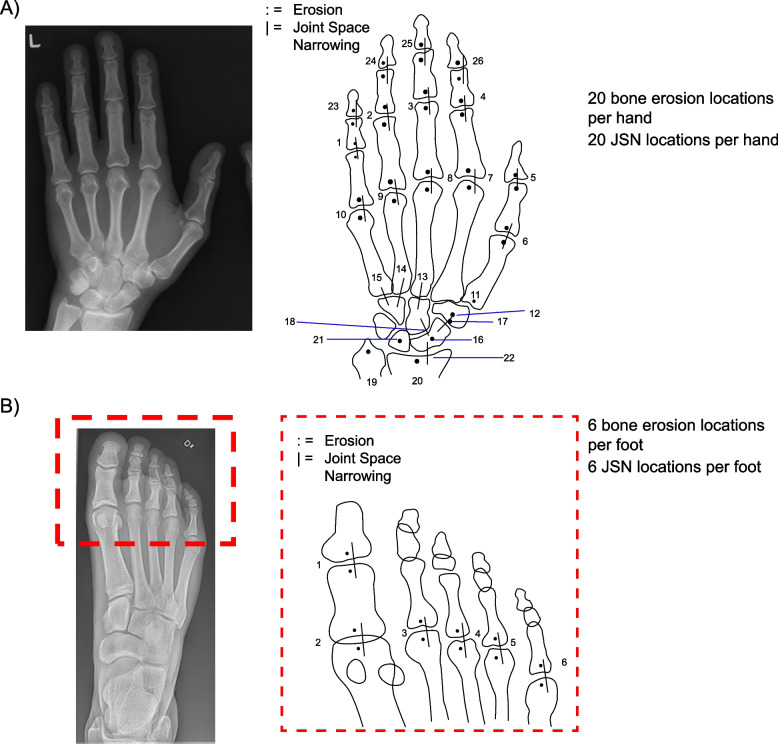
Radiographical scores of erosion and joint space narrowing in **A** hand and **B** foot. JSN, joint space narrowing

RD of hands/wrists/feet in patients with PsA was assessed using the vdH-modified Sharp scores for JSN and bone erosion, where the scores for bone erosion (0–5 in the hands and 0–10 in the feet) and JSN (0–4) were summed [13]. A total of 20 each bone erosion locations and JSN locations was assessed per hand, and a total of 6 each was assessed per foot (Fig. 1). The maximum erosion score for all 40 hand locations was 200, and for all 12 feet locations was 120. The total possible score for erosion was 320, and JSN was 208. The possible total radiographic score ranged from 0 to 528 (both hands and feet combined, where higher scores indicated greater joint damage).

Joint activity was assessed in terms of joint tenderness (yes/no) and swelling (yes/no). The number of tender and swollen joints were summed, separately, over the set of joints identified by vdH-modified Sharp locations but notice that a single tender or swollen assessment on a wrist was linked to the average of six RD JSN or bone erosion scores over the wrist locations. Therefore, for these analyses, the joint tender and swollen counts (TJC, SJC) were summed over 42 “joints” (28 in the hands, 2 “averaged joints” in the wrists, and 12 joints in the feet). See Fig. 1 for specific combined locations on and near the wrist.

To assess the extent of RD among patients at baseline, prevalence of pre-existing RD was defined in terms of the proportion of patients with joint erosion and JSN radiological scores exceeding various thresholds (0, 1, 1.5, and 2); prevalence for each RD type (erosion, JSN) was summarised over groups of patients with time since PsA diagnosis < 2, 2–5, 5–10, and > 10 years.

To assess the association between RD and joint activity at baseline, two approaches were taken: one, referred hereafter as patient-level, quantified RD by the vdH-mTSS (single score per patient) and joint activity by the TJC and SJC (single quantities per patient). A second approach, referred hereafter as the joint-level approach, quantified RD by the vdH-modified Sharp JSN and bone erosion scores at each joint and quantified the joint activity by the binary tender and swollen (yes/no) scores. For the wrists, single joint activity scores (tender yes/no and swollen yes/no) were mapped to the average of multiple vdH-modified JSN and bone erosion scores in 6 locations each associated with the wrists (see Fig. 1: JSN locations: carpometacarpal 3, 4, and 5, radiocarpal, multangular-navicular, and capitate-navicular-lunate; and bone erosion locations: first metacarpal, distal radius, distal ulna, trapezoid-trapezium, navicular, and lunate).

To assess the influence of pre-existing RD on secukinumab therapy, treatment response was assessed using SJC/TJC at weeks 0, 16, and 52 and the proportion of patients achieving minimal disease activity (MDA) over time (in FUTURE 5 only). MDA was assessed on a response of at least five of the following seven items: ≤ 1 TJC, ≤ 1 SJC, Psoriasis Area and Severity Index (PASI) ≤ 1 or body surface area ≤ 3%, patient pain visual analogue score (VAS) ≤ 15, patient global assessment of disease activity VAS ≤ 20, Health Assessment Questionnaire Disability Index (HAQ-DI) ≤ 0.5, and tender entheseal points ≤ 1.

### Statistical analysis

The association between the prevalence of pre-existing RD at baseline and time since PsA diagnosis was investigated through visualisation of JSN and bone erosion prevalence (as defined by each threshold value *T* = 0, 1, 1.5, 2) in groups of patients with time since PsA diagnosis in the time subintervals < 2, 2–5, 5–10, and > 10 years.

The association between RD and joint activity prior to secukinumab therapy was investigated at the patient- and joint-level. Thus, the association between the two patient-level RD scores (JSN and bone erosion) and the two patient-level joint activity scores (tenderness and swelling) were investigated through visualisation and quantified by Pearson correlation.

Mixed-effects logistic regression models were fit to tender/swollen data to quantify the association at baseline between overall RD in hands, wrists, and feet, on a patient-level and on an individual joint-level. Random effects were used to account for variability at both levels of data grouping, namely, within patients and joints-within-patients.

Logistic regression for visualisation and mixed-effect generalised linear models for inferences were used to estimate the probability of joint activity as a function of RD at each joint across all patients. The intercept coefficient from the logistic regression represents the log odds of having tender/swollen joints when the RD is 0 at the specific joint. The slope coefficient from logistic regression represents the increased risk of having a tender/swollen joint as RD increases (i.e., the log odds ratio of tenderness increase as 1 unit of RD score increases). A hypothesis of no association between disease activity and RD score was performed at each joint. For visualisation, the log odds ratios were rescaled to simple probabilities and the probability of joint activity (tender, swollen) was plotted versus values of baseline RD scores for each joint; the full set of all joint plots was displayed in a layout reminiscent of hands and feet.

Four associations between pre-existing RD and secukinumab response were assessed using the two vdH-modified Sharp JSN and bone erosion scores and the two joint activity scores TJC and SJC at weeks 0 (baseline), 16 and 52 conditioned on secukinumab doses 150 mg (NL) and 150 mg and 300 mg. Due to space limitations, only the two displays for TJC as functions of RD scores JSN and bone erosions are shown in this paper, and the remaining two displays for SJC as functions of RD scores JSN and bone erosions may be found in the supplement material. Each visualisation display includes three panels, one per each secukinumab dose, and each panel contains a scatter plot of TJC versus the baseline RD JSN or bone erosion as recorded at weeks 0, 16, and 52 with plotting symbols denoting the visit time. Loess smoothed curves were computed at each visit time and added to depict the average trend of TJC as functions of JSN and bone erosion scores at each time point.

Similarly, patient-level treatment response was assessed in the proportion of patients achieving MDA over all assessments within 1 year, where FUTURE 5 data of 987 patients were analysed. The MDA chance of remission was investigated by different vdH-mTSS scores at baseline. The response was plotted longitudinally with pooled secukinumab doses and patients grouped based on low or moderate versus high vdH-mTSS, as per baseline vdH-mTSS tertiles.

## Results

**Fig. 2:**
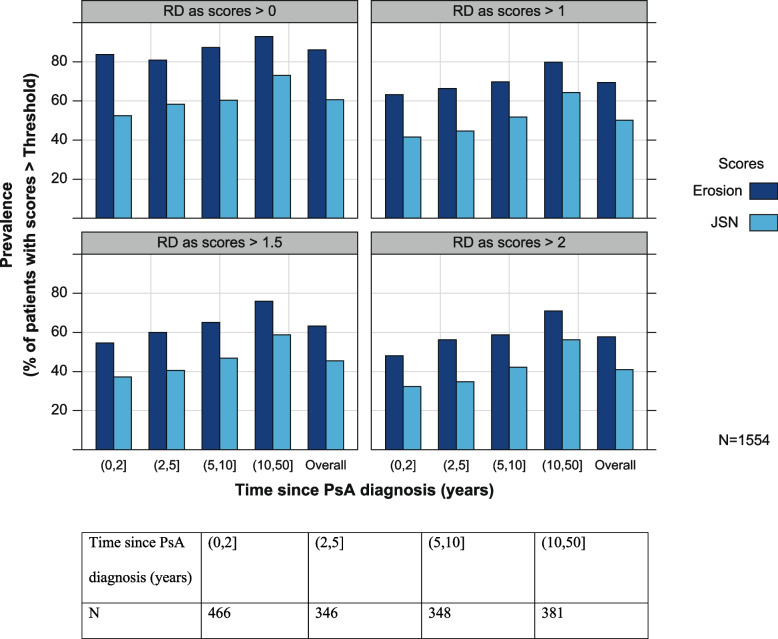
Prevalence of erosion and joint space narrowing at baseline in patients with psoriatic arthritis. Each panel depicts prevalence of erosion and JSN at baseline as a function of PsA duration categories. Prevalence definition (i.e., when a patient may be declared to have joint damage or not) may be quantified according to different threshold values of the erosion and JSN radiological scores. Yet the patterns of higher erosion than JSN prevalence and increasing RD for patients with longer PsA duration are consistent for all threshold values. JSN, joint space narrowing; *N*, total number of patients; PsA, psoriatic arthritis; RD, radiographical damage

Prevalence estimates of erosion and JSN at baseline in patients with PsA are given in Fig. 2. A substantial level of erosion and JSN at baseline was observed in these patients, and most patients reported some degree of erosion by the time they were diagnosed with PsA. Furthermore, a larger proportion of patients presented with erosion than with JSN. Many patients with a PsA diagnosis of < 2 years already had some erosion damage, and JSN to a lesser extent, even when higher thresholds of 2 were considered.

Patients with longer time since diagnosis of PsA (5–10 or 10–50 years) experienced a higher prevalence of RD as assessed by JSN and bone erosion. Erosion prevalence was higher than JSN prevalence across all groups of PsA time since diagnosis.

**Fig. 3:**
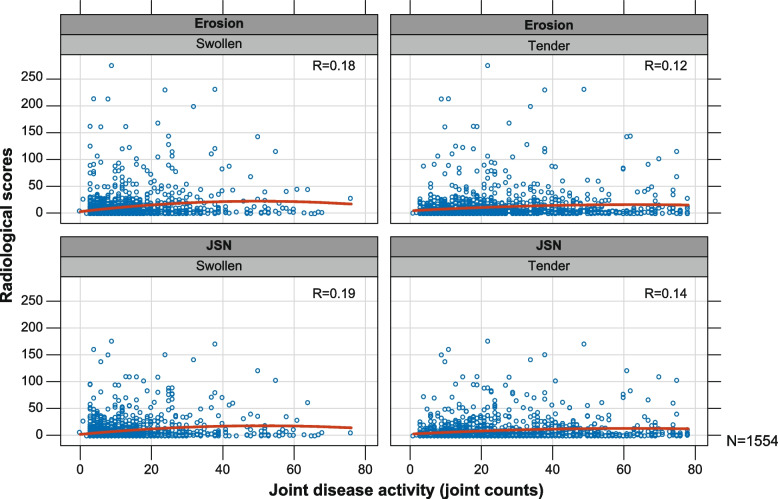
Radiographical scores and joint activity counts (patient-level data). Each panel depicts the relation (or lack thereof) between radiographic scores and joint disease activity counts. The headings at the top of each panel indicate the scores (points) being shown, e.g., erosion and swollen counts. The R values depict the Pearson correlation coefficients, and the red curves depict a rather weak trend over the point clouds. JSN, joint space narrowing; *N*, total number of patients; R, Pearson correlation coefficient

A weak association, as assessed by Pearson correlation, was observed between joint activity (TJC, SJC) and structural damage (erosion, JSN) at the patient-level (Fig. 3). The Pearson correlation coefficient for the number of swollen joints versus erosion was 0.18, and swollen joints versus JSN was 0.19; similarly, the correlation coefficient for tender joints versus erosion was 0.12, and tender joints versus JSN was 0.14. However, a strong association was found between joint activity and RD at the individual joint-level, with a higher probability of tender and swollen joints being associated with higher JSN and erosion scores (of the 42 analysed joints; all showed statistical significance at the unadjusted 0.05 level for the relationship between joint tenderness [yes/no] and its JSN score, all but one for tenderness and bone erosion scores, all but 2 for swollen and JSN scores, and all but 2 for swollen and bone erosion score).

**Fig. 4:**
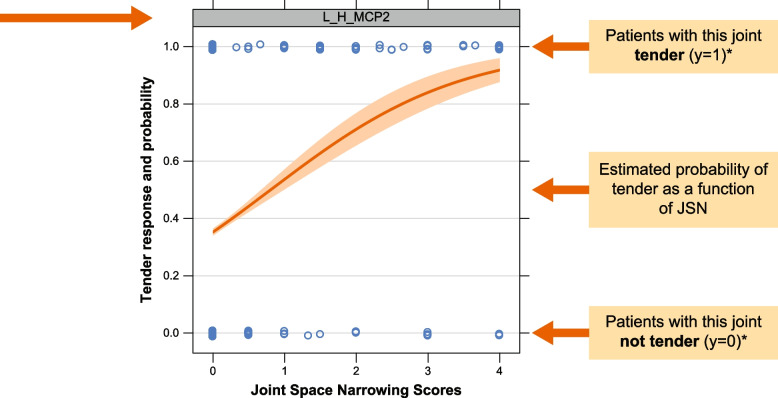
Probability of joint activity as a function of radiographic damage. The plot depicts the probability of tenderness as a function of JSN score at baseline. The curve was computed using logistic regression and re-scaled to a probability scale. The slope estimates the increased probability of tenderness for a unit increase of JSN score, while the *y*-intercept estimates the expected proportion of tender L_H_MCP2 joints in the absence of JSN (JSN = 0) among these patients. The JSN scores (0 = low,..., 4 = high) were plotted on the *x*-axis and tender scores (0 = no, 1 = yes) on the *y*-axis. *For *y* = 0, the patient does not have tenderness at this joint, while for *y* = 1, the patient has tenderness at this joint. JSN, joint space narrowing; L_H_MCP2, left hand metacarpo-phalangeal finger 2

**Fig. 5:**
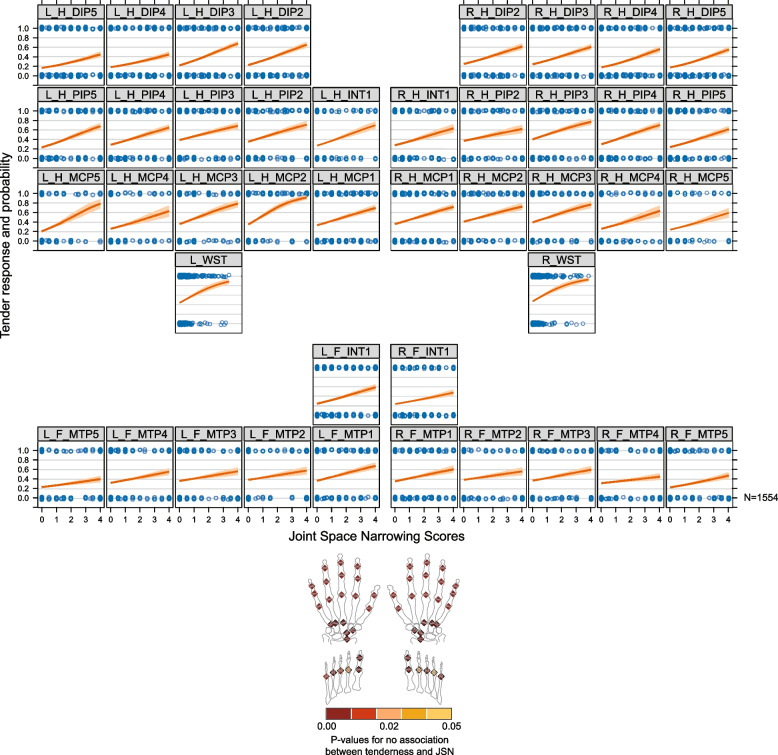
Probability of joint tenderness for values of joint space narrowing in individual joints of hands, wrists, and feet. Panel positions on the main plot suggest the position of joints in hands and toes (colours in the anatomical diagram and its legend depict the *p*-values for test of no association between tenderness and JSN in the corresponding panel (joint) of the main plot). JSN, joint spare narrowing; *N*, total number of patients

Figure 4 shows, as an example, the probability of joint activity as a function of RD JSN in the single left hand metacarpal index finger 2 (MCP2) joint. The curve represents the probability that a randomly selected patient from this population will experience tenderness at the corresponding JSN RD score, i.e., the values along the *x*-axis. The intercept in the plot implies that even when such a randomly chosen patient does not have detectable JSN, there was prevalence of tenderness at the left hand MCP2 joint. For example, for the lowest score of JSN, the probability of tenderness (*y*-axis) is close to 0.4 which is about a 40% chance of experiencing tenderness at this joint for a JSN score close to 0. For a higher baseline RD JSN score, the probability of tenderness was increasing, and for a patient with a RD JSN score of about 4, the probability of experiencing tenderness was about 90% at this joint. The slope, which varies across the values of JSN, depicts the increase of JSN probability per unit of JSN increase. The prevalence of tenderness increases substantially with increases in JSN. These results clearly identify a stronger association at the joint-level, particularly at baseline (the *P*-value on the test of no association between TJC and RD JSN was less than 0.001). A dynamic inter-dependence was observed between disease activity and structural damage. It was observed that for all joints, probability of tenderness increases with higher JSN (Fig. 5), all joints showed (unadjusted) statistical significance of *P* < 0.05 for test of no-association. The probability of joint tenderness and erosion values is shown in supplementary figure S1.

The probability of joint activity TJC as a function of RD bone erosion at baseline and after 52 weeks of secukinumab treatment for one joint is shown in supplementary figure S2, and for hands, wrists, and feet is presented in supplementary figure S3.

**Fig. 6:**
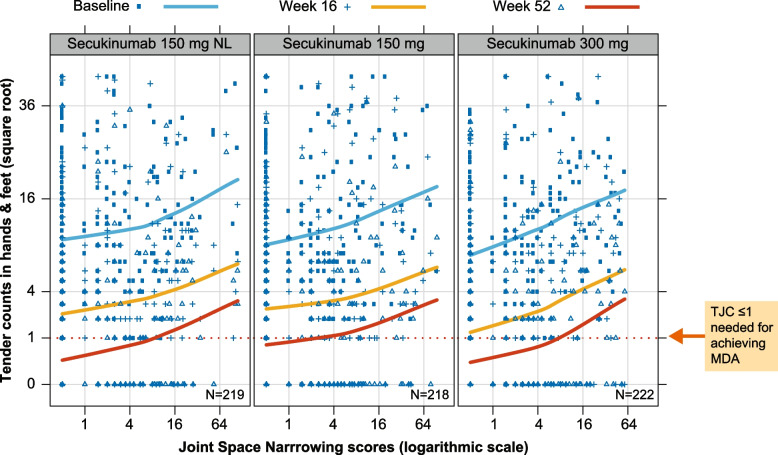
TJC in association with JSN score by secukinumab treatment and visits. This analysis is based only on FUTURE 5, since FUTURE 1 deals with unapproved doses. Each panel depicts a scatterplot of joint tender counts versus JSN vdH-mTSS scores at three time points (Weeks 0, 16, and 52) for each secukinumab dose. The loess smoothed curves depict the overall pattern of TJC and JSN at each time point, and thus the overall improvement in terms of joint counts over time due to secukinumab treatment. However, patients with high radiographical JSN scores tend to have incomplete recovery even after 52 weeks of treatment. JSN, joint space narrowing; MDA, minimal disease activity; NL, no loading doses; TJC, tender joint count

**Fig. 7:**
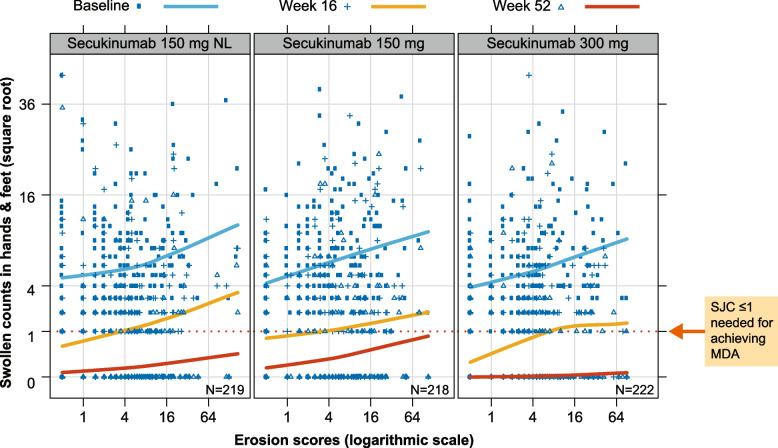
SJC in association with erosion score by secukinumab treatment and visits. This analysis is based only on FUTURE 5, since FUTURE 1 deals with unapproved doses. Each panel depicts a scatterplot of joint swollen counts versus erosion scores at three time points (weeks 0, 16, and 52) for each secukinumab dose. The loess smoothed curves depict the overall pattern of SJC and erosion at each time point, and thus the overall improvement in terms of joint counts over time due to secukinumab treatment. Unlike tender joint counts, swollen counts for patients with high radiographical erosion scores do tend to reach more complete remission. MDA, minimal disease activity; *N*, total number of patients; SJC, swollen joint count; NL, no loading doses

Secukinumab (150 mg, 150 mg NL, and 300 mg) reduced TJC across all values of JSN at week 16 and week 52 (Fig. 6), but patients with highest RD had a reduced likelihood of achieving complete resolution (i.e., TJC = 0). Similarly, secukinumab 150 mg, 150 mg NL, or 300 mg reduced SJC across all values of erosion scores up to week 52 (Fig. 7), but patients with highest RD had a reduced likelihood of achieving complete resolution (i.e., SJC = 0). It should be noted that for the highest dose of secukinumab (i.e., 300 mg), the average SJC curves lies completely below score of 1 at week 52.

**Fig. 8:**
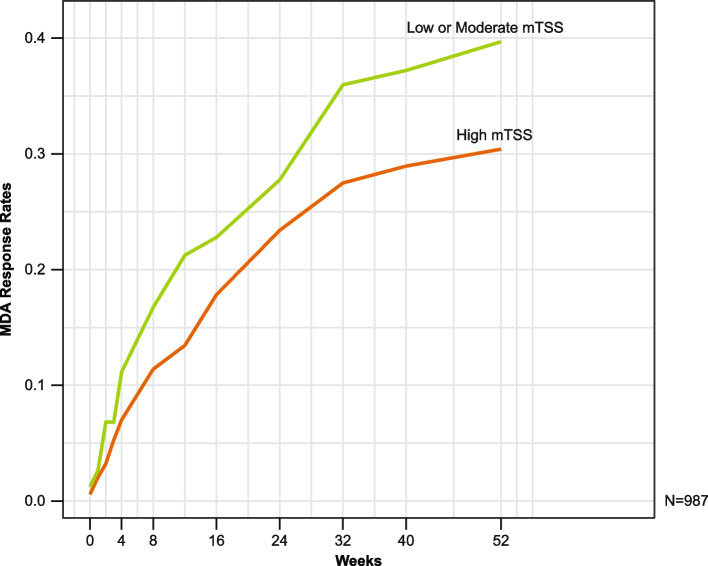
Radiographic damage as per patient-level mTSS and MDA over 52 weeks of secukinumab treatment. MDA, minimal disease activity; mTSS, modified total Sharp score; *N*, total number of patients

MDA response rates against RD at the patient-level mTSS are presented in Fig. 8. All secukinumab doses were pooled for this analysis and plotted after splitting the patients according to mTSS (low or moderate, score ≤ 8, 66% of patients; or high, score > 8, 33% of patients). Patients with high mTSS had a lower chance of reaching MDA with secukinumab treatment up to week 52.

## Discussion

It has been well-established in PsA that identification of predictors for the progression of joint damage clinically and radiographically would lead to better management of the disease in these patients [14]. It is evident from studies that an association exists between the number of swollen joints and progression of radiological damage, and the risk for progression of joint damage increases with an increase in the number of joints that were previously damaged [14]. Radiographic assessment of joint damage in inflammatory arthritis and degree of inhibition with treatment is typically represented as a summary score of erosion and JSN of all joints assessed.

This post hoc analysis of two large phase 3 trials of secukinumab in PsA quantified the prevalence and magnitude of pre-existing RD at both a patient and specific joint level at baseline, the association between RD and SJC and TJC prior to secukinumab therapy, and the effect of RD on response to secukinumab treatment at weeks 16 and 52. At baseline, patients with PsA in our pooled data showed substantial prevalence of RD (erosion and JSN) which was related to time since diagnosis. The pre-existing RD was weakly associated with joint activity when estimated at the overall patient-level but strongly associated when estimated within-patient individual joints.

To supplement the patient-level approach, the analysis was extended to joint-level, the joint activity measurements (i.e., tenderness and swelling) were mapped one-to-one to the RD scores according to anatomical locations in hands, wrists, and feet. Studies [13, 14] have shown that in patients with PsA, progression of joint damage is linked to joint disease activity and damage of that particular joint. Furthermore, the presence of joint tenderness increases the risk of joint damage even in the absence of joint swelling [15]. A prospective study in PsA showed joints with inflammation, especially swollen joints, predicted the progression of RD and that active joint inflammation could lead to joint damage [14]. The current analysis shows a converse relationship: joint disease activity itself is linked to joint damage, thus suggesting an inter-dependence of inflammatory joint processes and radiological joint damage, each linked to the other.

For individual joints, the probability of joint tenderness increased with higher radiographic scores at baseline—this association is greatly diluted and sometimes underestimated if analysed using traditional patient-level summaries; it was estimated that substantially increased odds-ratios of tenderness or swelling for unit-increases in JSN and erosion values differentially in hands, wrists, and feet (53%, 109%, and 22% for hand, wrist, and foot joints, respectively).

Prevention of joint damage is one of the major goals of PsA treatment and physicians ideally aim to achieve complete remission in the joints. Achievement of complete inhibition of tenderness and swelling was associated with secukinumab therapy in a dose dependent manner. Higher RD at baseline was associated with a decreased likelihood of complete inhibition of joint tenderness and swelling with secukinumab therapy. Similarly, patients with the highest RD at baseline were less likely to achieve MDA with secukinumab therapy. It was assumed that the association between RD and MDA could be through the association between RD and SJC/TJC. A limitation of this study is that the analysis is based on cross-sectional data, and not on a complete follow-up of patient journeys. Hence, clear statements cannot be made about whether erosion manifests before JSN in patients with PsA, even though a higher prevalence of erosion than JSN was observed in these patients cross-sectionally.

## Conclusions

Patients with PsA at baseline of both phase 3 studies of secukinumab showed substantial prevalence of RD that was related to time since diagnosis. Furthermore, pre-existing RD was weakly associated with joint activity at the overall patient-level but strongly associated with patient’s individual joint status. Secukinumab therapy, in a dose dependent manner, was associated with inhibition of joint tenderness and swelling, although high RD at baseline was associated with reduced likelihood of full inhibition.

## Supplementary Information

**Additional file 1: Supplementary figure S1.** Probability of joint tenderness for values of erosion in individual joints of hands, wrists, and feet. **Supplementary figure S2.** Probability of joint activity as a function of radiographic damage at baseline and after 52 weeks of secukinumab (any dose) for one joint. **Supplementary figure S3.** Probability of joint activity as a function of radiographic damage at baseline and after 52 weeks of secukinumab (any dose).

## Data Availability

The data sets generated during and/or analysed at the end of the current study are not publicly available. Novartis is committed to sharing with qualified external researchers’ access to patient-level data and supporting clinical documents from eligible studies. These requests are reviewed and approved based on scientific merit. All data provided are anonymised to respect the privacy of patients who have participated in the trial in line with applicable laws and regulations. The data may be requested from the corresponding author of the manuscript.

## Abbreviations

PsA: Psoriatic arthritis
JSN: Joint space narrowing
QoL: Quality of life
IL: Interleukin
TNFi: Tumour necrosis factor inhibitor
RD: Radiographic damage
SJC: Swollen joint count
TJC: Tender joint count
NL: Without loading dose
vdH-mTSS: van der Heijde-modified total Sharp score
MDA: Minimal disease activity
VAS: Visual analogue scale
HAQ-DI: Health Assessment Questionnaire Disability Index
