# Supplementary material for: Quantification of pre-existing radiographic damage and its relationship with joint activity and long-term clinical outcomes with secukinumab therapy in patients with psoriatic arthritis
Source: Arthritis Res Ther. 2022 Dec 28;24:283. doi: 10.1186/s13075-022-02944-1 (PMC9795644; doi:10.1186/s13075-022-02944-1)
Supplement: Supplementary file 1 — Additional file 1: Supplementary figure S1. Probability of joint tenderness for values of erosion in individual joints of hands, wrists, and feet. Supplementary figure S2. Probability of joint activity as a function of radiographic damage at baseline and after 52 weeks of secukinumab (any dose) for one joint. Supplementary figure S3. Probability of joint activity as a function of radiographic damage at baseline and after 52 weeks of secukinumab (any dose). [file 13075_2022_2944_MOESM1_ESM.docx]

**Supplementary file**


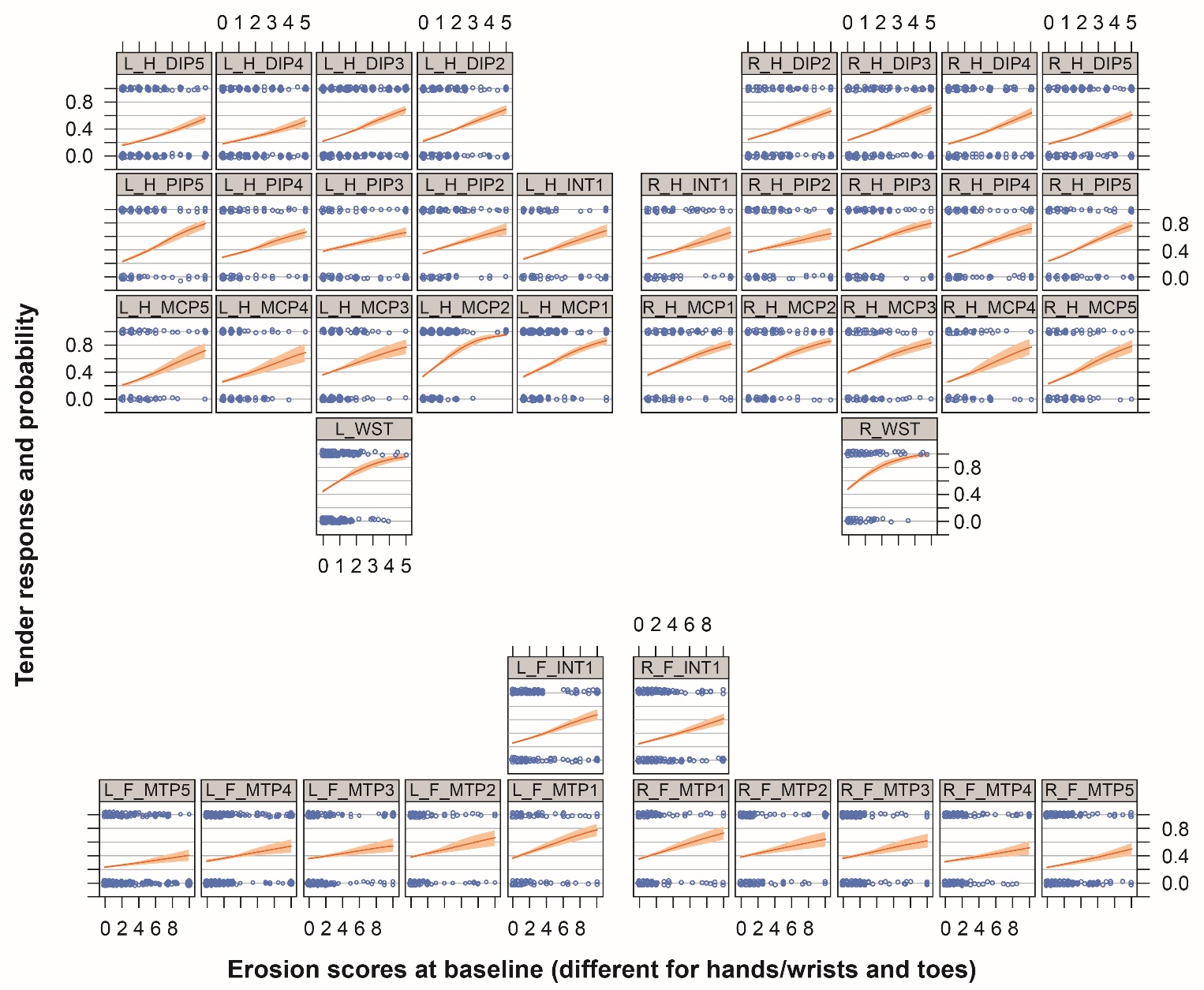
**Supplementary figure S1. Probability of joint tenderness for values of erosion in individual joints of hands, wrists, and feet**

Erosion scores for hands and wrists can have a maximum of 5, while scores for toes can go up to 10

**Supplementary figure S2. Probability of joint activity as a function of radiographic damage at baseline and after 52 weeks of secukinumab (any dose) for one joint**


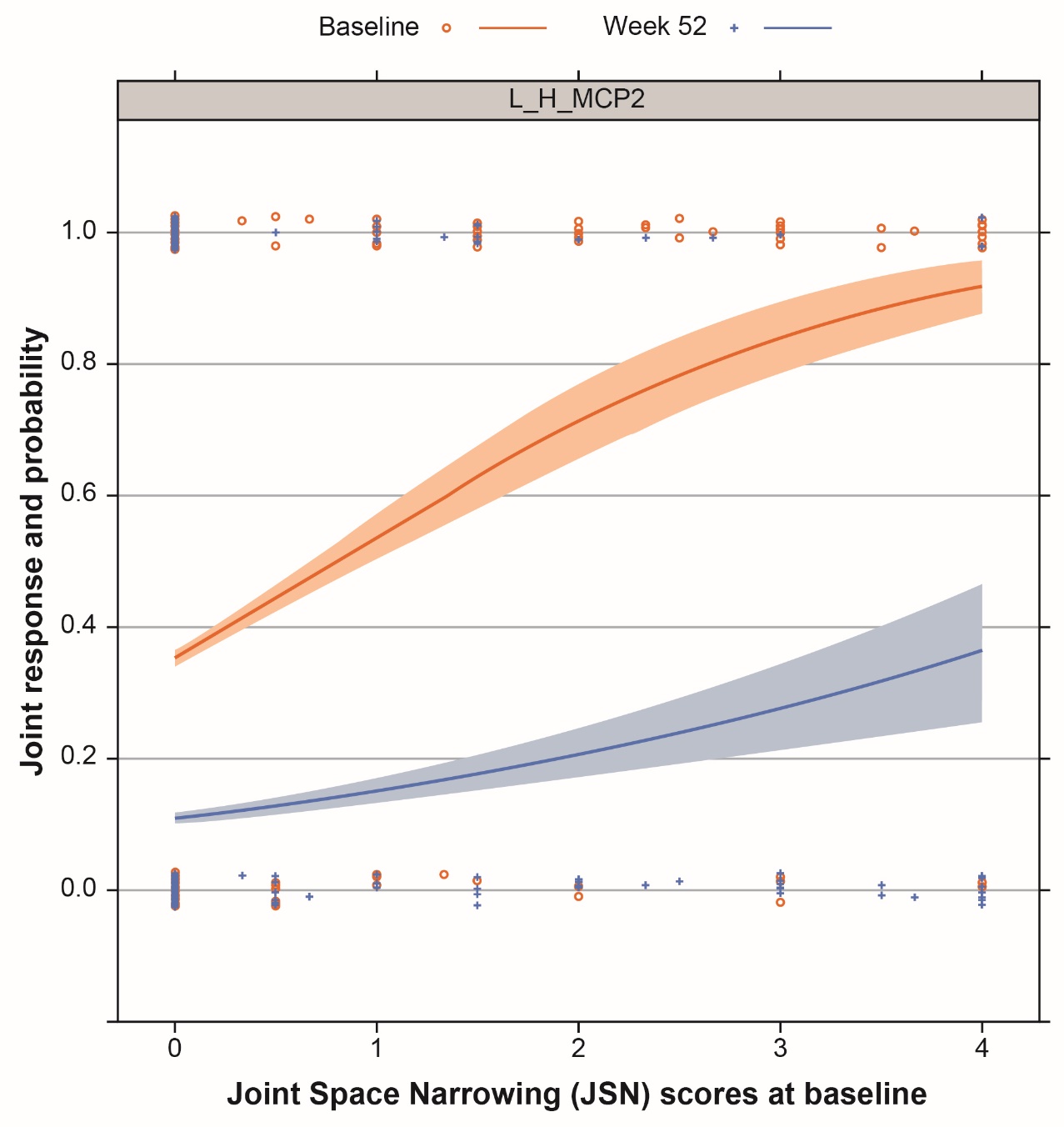
 L_H_MCP2, left hand metacarpo-phalangeal finger 2

**
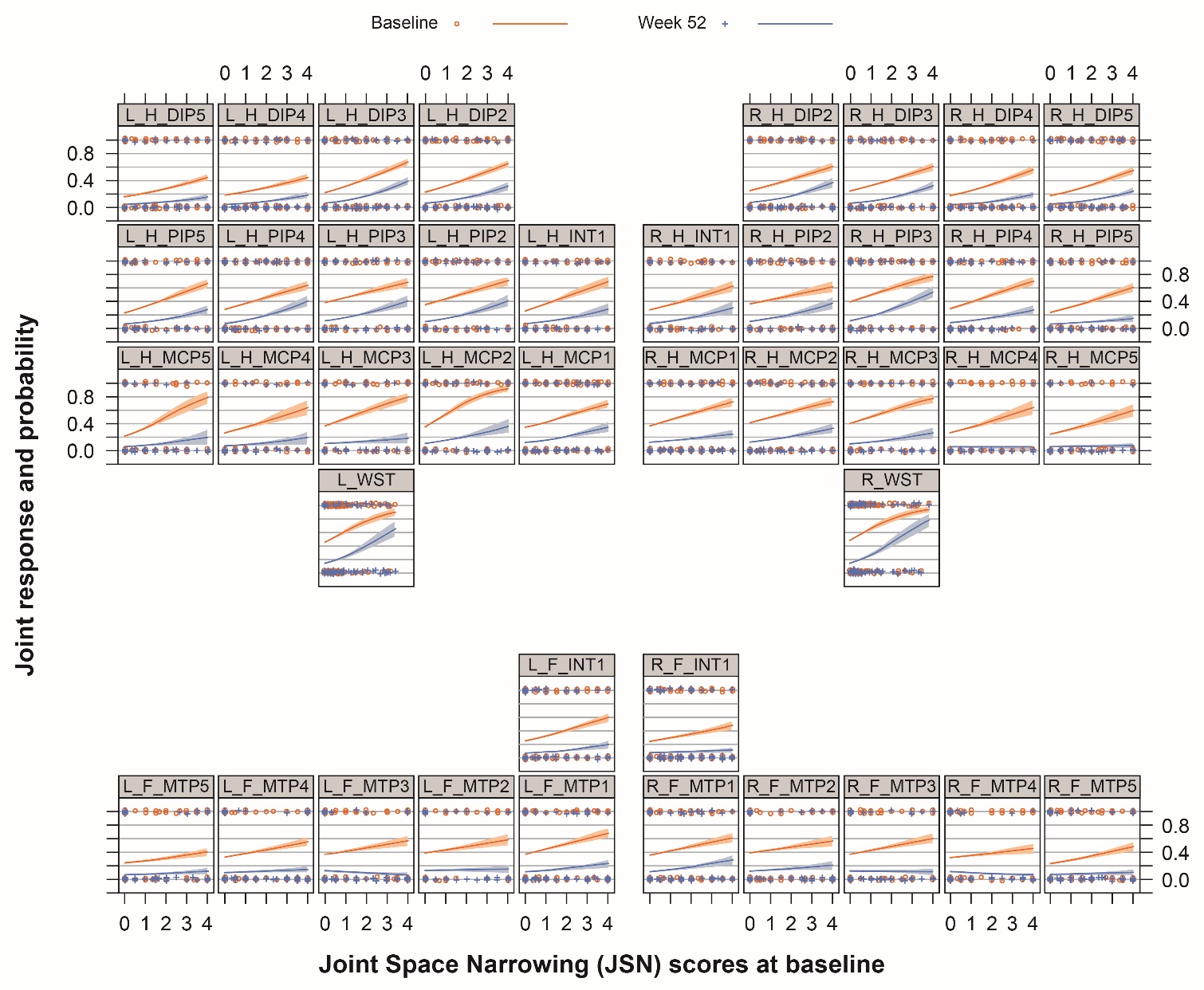
Supplementary figure S3. Probability of joint activity as a function of radiographic damage at baseline and after 52 weeks of secukinumab (any dose)**
